# RSV testing practice and positivity by patient demographics in the United States: integrated analyses of MarketScan and NREVSS databases

**DOI:** 10.1186/s12879-022-07659-x

**Published:** 2022-08-08

**Authors:** Phuong T. Tran, Sabina O. Nduaguba, Vakaramoko Diaby, Yoonyoung Choi, Almut G. Winterstein

**Affiliations:** 1 Department of Pharmaceutical Outcomes and Policy, College of Pharmacy, University of Florida, 1225 Center Drive, PO Box 100496, Gainesville, FL 32611-0496 USA; 2 Center for Drug Evaluation and Safety, University of Florida, Gainesville, FL USA; 3 Faculty of Pharmacy, HUTECH University, Ho Chi Minh City, Vietnam; 4 Department of Pharmaceutical Systems and Policy, School of Pharmacy, West Virginia, Morgantown, WV USA; 5 West Virginia University Cancer Institute, Morgantown, WV USA; 6 Global Value and Real-World Evidence, Otsuka America Pharmaceutical, Inc., Princeton, NJ USA; 7 Center for Observational and Real-World Evidence, Merck & Co., Inc., Kenilworth, NJ USA; 8 Department of Epidemiology, College of Medicine and College of Public Health and Health Professions, University of Florida, Gainesville, FL USA

**Keywords:** RSV, Respiratory syncytial virus, Antigen test, PCR test, Viral culture, Antibody test, CDC’s National Respiratory and Enteric Virus Surveillance System, RSV epidemiology, RSV measurement, Automated healthcare data

## Abstract

**Background:**

RSV-incidence estimates obtained from routinely-collected healthcare data (e.g., MarketScan) are commonly adjusted for under-reporting using test positivity reported in national Surveillance Systems (NREVSS). However, NREVSS lacks detail on patient-level characteristics and the validity of applying a single positivity estimate across diverse patient groups is uncertain. We aimed to describe testing practices and test positivity across subgroups of private health insurance enrollees in the US and illustrate the possible magnitude of misclassification when using NREVSS to correct for RSV under ascertainment.

**Methods:**

Using billing records, we determined distributions of RSV-test claims and test positivity among a national sample of private insurance enrollees. Tests were considered positive if they coincided with an RSV-diagnosis. We illustrated the influence of positivity variation across sub-populations when accounting for untested acute respiratory infections.

**Results:**

Most tests were for children (age 0–4: 65.8%) and outpatient encounters (78.3%). Test positivity varied across age (0–4: 19.8%, 5–17: 1.8%, adults: 0.7%), regions (7.6–16.1%), settings (inpatient 4.7%, outpatient 14.2%), and test indication (5.0–35.9%). When compared to age, setting or indication-specific positivity, bias due to using NREVSS positivity to correct for untested ARIs ranged from − 76% to 3556%.

**Conclusions:**

RSV-test positivity depends on the characteristics of patients for whom those tests were ordered. NREVSS-based correction for RSV-under-ascertainment underestimates the true incidence among children and overestimate rates among adults. Demographic-specific detail on testing practice and positivity can improve the accuracy of RSV-incidence estimates.

**Supplementary Information:**

The online version contains supplementary material available at 10.1186/s12879-022-07659-x.

## Background

Respiratory syncytial virus (RSV) is one of the most common causes for significant respiratory illness in young children and older adults [1, 2]. Due to limited options for treatment and immunoprophylaxis [3], and a common under-appreciation of the importance of RSV for some patient populations [4–6], RSV testing is not routinely recommended and often not performed [4–6]. Real-world population-based data on RSV epidemiology (e.g., RSV incidence and seasonal pattern) is therefore dependent on testing practices and RSV incidences often underestimated due to lack of testing. Thus, and missing information on RSV testing practices to inform measurement of RSV epidemiology has been identified as significant gap by the Centers for Disease Control and Prevention (CDC) [7].

To estimate RSV incidence, public health agencies rely on prospective studies that include comprehensive testing for pathogens but that are often limited by small sample size, especially in adults [8–10] and short study periods, resulting in insufficient capture of high-risk groups [8–11]. To overcome these issues and to obtain population-based RSV incidence estimates from real-world data that can capture national trends over multiple seasons, statistical models have been used that adjust for RSV under-ascertainment. These approaches model the proportion of positive tests per week and region reported in CDC’s National Respiratory and Enteric Virus Surveillance System (NREVSS) against infection incidence estimates, typically lower respiratory tract infections (LRTI), obtained from routinely-collected healthcare encounter diagnoses, which often do not include detail on the pathogen (due to lack of testing or lack of coding) [12–15]. The accuracy of such estimates relies on the assumption that the laboratory-reported tests in NREVSS (and thus, the proportion of positive tests) are representative of the population with LRTIs for which RSV incidence is to be measured. However, limited information in NREVSS about the submitted specimens available for public use (e.g., no information about patient age, clinical setting, or type or severity of the infection) raises concerns about the validity of this assumption, because testing practice and percent of positive tests may vary across patient groups and settings. Moreover, NREVSS is a voluntary, passive system, which may not provide a representative sample of RSV tests nationwide. Finally, the four available types of RSV tests, including polymerase chain reaction (PCR), viral culture, immunofluorescence, and rapid antigen vary in sensitivity and specificity, availability, turnover time, and cost [16–20]. This can result in different choices of test types across clinical settings and patient populations, influencing overall RSV positivity and thus, incidence estimates.

Accurate assessment of the disease burden is essential for public health efforts [7].We aimed to describe testing practices and test positivity across various patient subgroups obtained from a large national sample of privately insured patients’ billing records to inform on the applicability of NREVSS data for RSV incidence estimation. We further illustrated the possible magnitude of misclassification when using average population positivity rates in NREVSS to estimate RSV incidence for certain subpopulations.

## Methods

### Data sources and study population

We used the IBM® MarketScan® Commercial Claims and Medicare Supplemental Databases from 2011 to 2019 to describe characteristics of children and adults enrolled in non-capitated health plans who were tested for RSV or had a medical encounter with an RSV diagnosis. MarketScan provides administrative claims data for employees and their dependents who are covered by employer-sponsored private health insurance in the US. Enrollees in capitated health plans were excluded because providers are reimbursed with lump sum payments rather then for individual services, which may compromise complete capture of medical encounters via claims and thus result in lower data quality). The MarketScan database provides patient-level longitudinal data for a national sample of more than 100 million enrollees in private insurance plans, including detail about their demographic characteristics, medical encounters, and dispensed prescription drugs. As with other national insurance claims databases, MarketScan has been used to derive population-based RSV incidence estimates [21–24].

For the same time period, we analyzed NREVSS to assess the concordance in test type distribution and test positivity with MarketScan. NREVSS is a voluntary surveillance system that collects aggregated weekly lab data from participating U.S. laboratories to monitor respiratory virus circulation [17, 25]. NREVSS provides quantity and results of diagnostic tests for RSV by test type (i.e., PCR, viral culture and antigen -a combination of rapid antigen and immunofluorescence) and by 10 regions classified by the U.S. Department of Health and Human Services (HHS) [25].

### Study design and inclusion criteria

For the assessment of RSV test distributions, we enrolled all children and adults in MarketScan who had one or more RSV tests from out- or inpatient settings. We identified RSV tests based on claims using Current Procedural Terminology (CPT) codes (Additional file 1: Table S1). Because some CPT codes for RSV testing are not specific [e.g., 87,798 Infectious agent detection by nucleic acid (DNA or RNA), not otherwise specified], we evaluated 3 alternative definitions to identify RSV tests, including a broad (i.e., all potential CPT codes), strict (i.e., specific CPT codes for RSV testing and nonspecific CPT codes accompanied by relevant diagnoses (e.g., a respiratory illness) on test claims (Additional file 1: Table S2)) and a very strict definition (i.e. only specific CPT codes specifying a test for RSV).

### Frequency of RSV tests

We estimated the frequencies of RSV tests in both MarketScan and NREVSS. In MarketScan, if multiple tests were conducted for the same patient on the same day (< 1%), the test with the highest sensitivity was retained (PCR > viral culture > antigen). Though an antibody test is not recommended to diagnose RSV infections, we report the proportion of antibody tests to fully capture testing practice. To be eligible for this category, we required patients to not have other RSV tests on the same day. NREVSS does not identify tests at the level of patients, thus only frequencies of tests are reported.

### Positivity of RSV tests

Test positivity was obtained directly from test results available in NREVSS. In MarketScan, we approximated the proportion of RSV positive tests based on diagnoses codes on laboratory claims or other medical encounters adjacent to the laboratory claims date, because the actual test result is not available from claims data. To do so, we searched for outpatient encounters with primary or secondary diagnosis codes indicating an RSV infection within ± 7 days of the RSV test claim or for inpatient encounters that overlapped with or followed an RSV test within 3 days. These time windows were chosen to accommodate turn-around times of RSV tests, which depend on test types and capacity for testing [20, 26, 27]. Patients were required to have continuous insurance coverage during these time windows to fully capture medical encounters.

We compared the test distributions and proportion of positive tests across strata estimated from claims data with those reported by NREVSS. The stratified analyses considered calendar year, quarter, HHS region and test type (all available in both databases) and age, assumed test indication and clinical setting in MarketScan. We obtained test indications from diagnoses codes on test claims and group diagnoses based on disease severity: we gave priority to infections with severe complications (i.e., septicemia/sepsis, respiratory distress/failure), followed by LRTIs, asthma exacerbations, upper respiratory tract infections (URTIs) including otitis, respiratory symptoms, and others (Additional file 1: Table S2).

For comparison of secular trends in testing, we standardized annual data (2011–2018) to the number of enrollee-years in 2019, because the MarketScan population changed over time due to different health plans providing data in each year. The size of the source population for the RSV tests in NREVSS is unknown.

### Influence of positivity variation on RSV incidence estimates

RSV test positivity rates are commonly used to estimate overall RSV incidence by imputing the presence of RSV among respiratory tract infections that carry no code for the responsible pathogen. To illustrate the impact of differences in the population from which test positivity rates are obtained and the population to which they are applied to estimate RSV incidence, we calculated the corrected RSV (cRSV) incidence. The total number of cRSV encounters was the sum of all acute respiratory infections with coded RSV (ARI_RSV_), which was directly obtained from MarketScan, plus the unknown number of ARIs without coded pathogen where the pathogen was likely RSV (${\overline{ARI} }_{RSV}$, Eq. 1).

1 $$\mathrm{cRSV \, incidence}=\frac{ {ARI}_{RSV} + {\overline{ARI} }_{RSV}}{\#\mathrm{ enrollee \, months}}$$

To obtain ${\overline{ARI} }_{RSV}$, we assumed that all ARIs without coded pathogen in MarketScan are a combination of ARIs with negative RSV test plus untested ARIs. The number of ARIs with negative RSV test was directly estimated from the RSV test positivity (e.g., if test positivity is 10% and we count 100 ARI_RSV_, the number of tested ARIs that were negative is 100/10%-100 = 900). Subtracting this estimated number of RSV-negative ARIs from all uncoded ARIs provided the number of untested ARIs. This number of untested ARIs was then multiplied with varying-test positivity rates (assuming 10%, 20%, 40%, 60%, 80%, or 100% of the positivity of observed RSV-tested ARIs) to obtain ${\overline{ARI} }_{RSV}$. This range reflects our assumption that positivity among untested ARIs is lower than that of tested ARIs because clinicians likely order a test for patients where RSV is suspected. An example to illustrate this calculation is presented in the Additional file 1: Supplementary Methods.

We compared the percent misclassification of corrected incidence rates across strata when using MarketScan stratum-specific (age, setting and indication) positivity rates versus the NREVSS average positivity rate (Eq. 2).

2 $$\mathrm{\%misclassification}=\frac{ cRSV \,incidence \,using \,NREVSS-cRSV \,incidece \,using \,MarketScan}{cRSV \,incidece \,using \,MarketScan}*100\%$$

All analyses were conducted for either antigen or PCR tests in each stratum. All analyses were conducted with SAS Studio 3.8 (SAS, Cary, NC).

## Results

From 2011 to 2019, NREVSS included 7 079 301 antigen, PCR, and viral culture tests for RSV diagnosis. The counts for RSV tests in MarketScan were 3 684 704 when using the broad, 1 471 777 for the strict and 1 206 704 for the very strict definition. When evaluating the trailing diagnosis codes on the same claims as our study CPT codes, we noted that the reduction in counts when using the broad versus the strict definition was mainly due to viral culture and PCR tests for sexually transmitted diseases. Requiring a diagnosis code for respiratory disease or symptoms to accompany the unspecified tests (strict definition) resulted in only a slight difference in counts when compared to the very strict definition and the distribution of test types looked more similar to NREVSS (Additional file 1: Table S3).Inspection of RSV positive cases also suggested that unspecified tests were commonly used and thus exclusion of these tests could have affected the observed test positivity. Thus, we decided to choose the strict definition to conduct all further analyses.

**Fig. 1:**
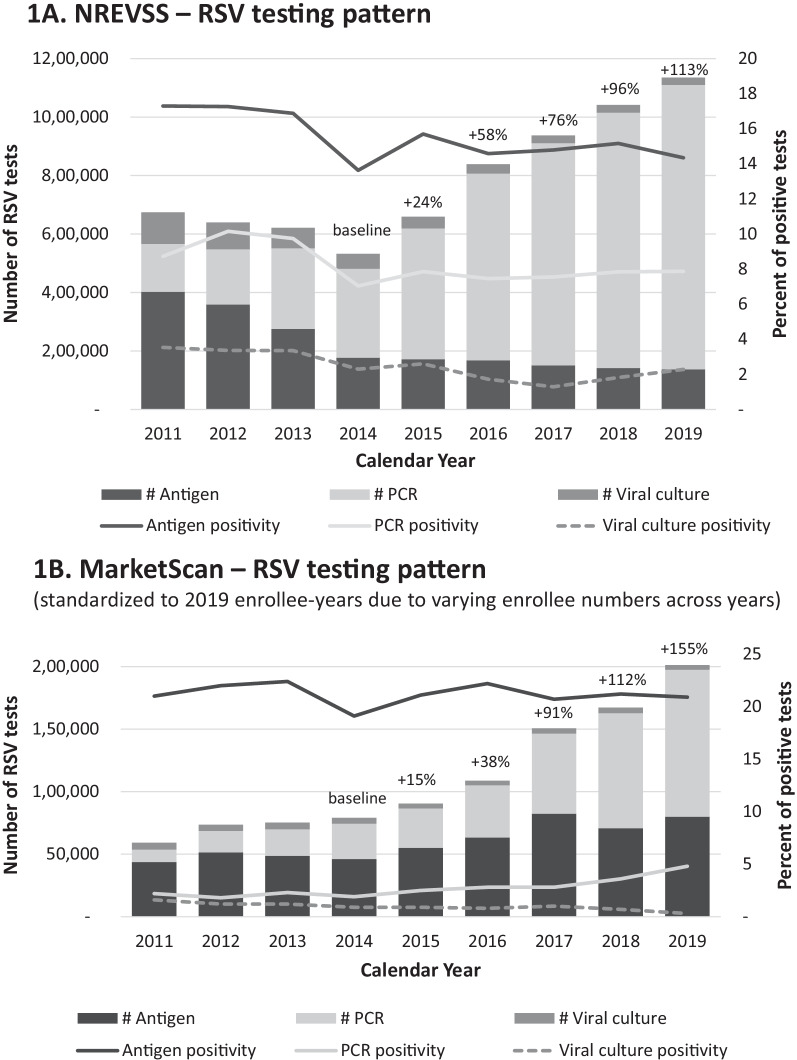
RSV test frequency and test positivity in MarketScan and NREVSS databases (2011–2019). In MarketScan RSV tests were identified using the “strict” definition, which required an RSV-specific test procedure code or an unspecific procedure code that was accompanied by a diagnosis indicative of respiratory illness (see Additional file 1: Table S1). Positivity was measured as the proportion of RSV tests accompanied by diagnoses codes on laboratory claims or adjacent medical encounters. Positivity for tests in NREVSS was directly extracted from recorded test results. RSV: respiratory syncytial virus; MarketScan: MarketScan Commercial Claims and Medicare Supplemental Databases; NREVSS: National Respiratory and Enteric Virus Surveillance System; PCR: polymerase chain reaction; #: number

The crude number of RSV tests increased from 2015 to 2019 in both NREVSS and MarketScan databases (Additional file 1: Table S3). This trend in MarketScan became more apparent after standardization to account for changes in the number of enrollees over time (Fig. 1). Consistent with seasonal patterns, the largest proportion of tests occurred in the first and fourth calendar quarter in both MarketScan and NREVSS. Examining demographic and clinical characteristics of tested patients in claims, which is not available in NREVSS, most of the tests were for infants and young children (age 0–4: 66.1%), conducted in outpatient settings (78.3%), and associated with diagnoses for URTI (31.9%) and respiratory symptoms (27.9%) (Additional file 1: Table S3). The strong association between age and RSV testing is illustrated by a testing incidence of > 6000 tests per 100,000 person-years among children less than 5 years of age and rates of less than 300 among all adult age groups combined.

The distribution of test types differed across the two databases with more antigen tests in claims. In MarketScan, we saw a slightly increased and relatively flat trend in number of antigen and viral culture tests, respectively, which contrasted with strongly declining numbers in NREVSS. The number of PCR tests increased in both databases (Fig. 1). About 97.7% of antigen, 32.5% viral culture and 53.3% PCR tests were ordered for children. While antigen tests were used mostly for patients with URTIs and LRTIs, PCR was used mostly for patients with URTIs or respiratory symptoms (Additional file 1: Table S4).

**Fig. 2:**
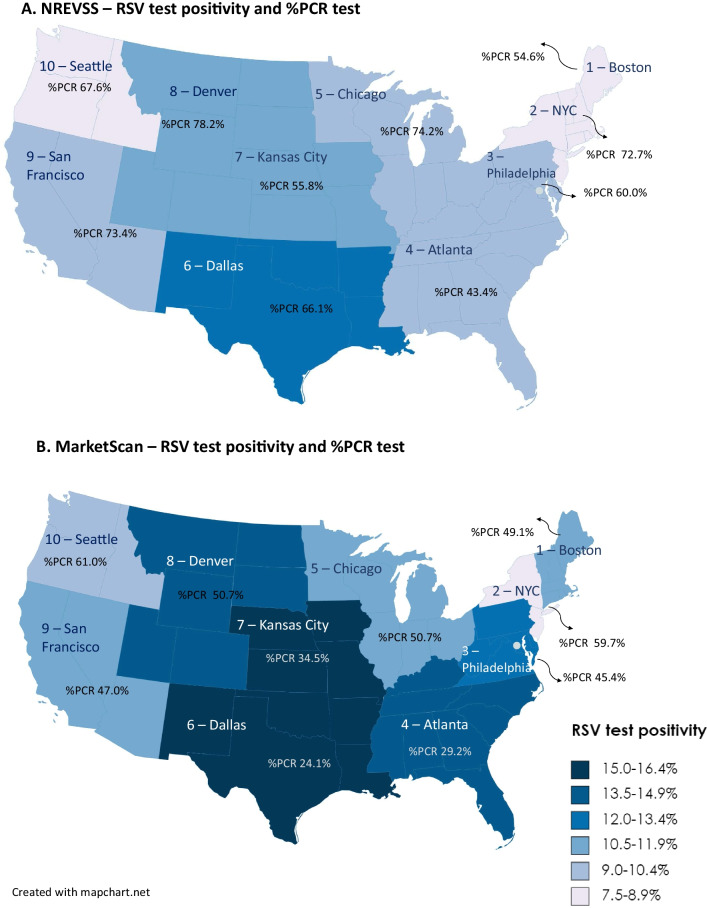
All-type RSV test positivity and percentage of PCR test among PCR, viral culture, and antigen tests in MarketScan and NREVSS (2011–2019) by HHS region. Notes: In MarketScan RSV tests were identified using the “strict” definition, which required an RSV-specific test procedure code or an unspecific procedure code that was accompanied by a diagnosis indicative of respiratory illness (see Additional file 1: Table S1). Positivity was measured as the proportion of RSV tests accompanied by diagnoses codes on laboratory claims or adjacent medical encounters. Positivity for tests in NREVSS was directly extracted from recorded test results. RSV: respiratory syncytial virus; MarketScan: MarketScan Commercial Claims and Medicare Supplemental Databases; NREVSS: National Respiratory and Enteric Virus Surveillance System; PCR: polymerase chain reaction; HHS region: the U.S. Department of Health and Human Services region

**Fig. 3:**
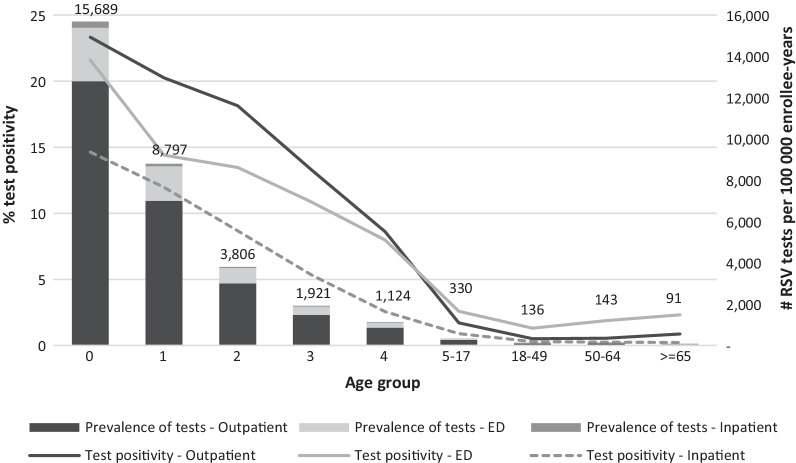
RSV testing frequency and positivity in MarketScan (2011–2019) by age group and clinical setting. In MarketScan RSV tests were identified using the “strict” definition, which required an RSV-specific test procedure code or an unspecific procedure code that was accompanied by a diagnosis indicative of respiratory illness (see Additional file 1: Table S1). Positivity was measured as the proportion of RSV tests accompanied by diagnoses codes on laboratory claims or adjacent medical encounters. Positivity for tests in NREVSS was directly extracted from recorded test results. RSV: respiratory syncytial virus; MarketScan: MarketScan Commercial Claims and Medicare Supplemental Databases; #: Number, ED: emergency department

RSV positivity was slightly higher in claims compared to NREVSS data across years, quarters, and HHS regions. Compared to NREVSS, MarketScan enrollees had higher positivity for antigen (21.2% versus 16.0%) but lower positivity for PCR tests (3.1% versus 7.9%) and viral cultures (1.1% versus 2.8%). Total test positivity in claims was 13.4%, with large variation across age groups (age range 0–4: 8.3–22.9%, 5–17: 0.4–3.0% and adults: 0.6–1.3%), regions (range 7.6–16.1%), clinical settings (inpatient 4.7%, ED 11.6%, and outpatient 14.2%), and indications for testing (range 5.0–35.9%) (Table 1). The southern and land-locked regions of the United States had higher positivity compared to the remaining regions in both MarketScan and NREVSS (Fig. 2). We observed a sharp drop of test positivity across all clinical settings from age 0 to 4 and of lesser magnitude thereafter. In adults, the test positivity remained relatively low and flat, though positivity increased slightly among older age groups (Fig. 3).

**Table 1:** RSV test positivity in MarketScan Commercial Claims and Medicare Supplemental Databases and the National Respiratory and Enteric Virus Surveillance System (2011–2019), total and stratified by tests type

Variables	Test positivity, %								
		MarketScan*					NREVSS		
	All tests (N = 1,418,020, 13.4%)	Antibody (N = 32,900, 18.5%)	Antigen (N = 786,065, 21.2%)	Viral culture (N = 66,696, 1.1%)	PCR (N = 532,359, 3.1%)	All tests (N = 7,079,301, 9.2%)	Antigen (N = 1,983,441, 16.0%)	Viral culture (N = 471,707, 2.8%)	PCR (N = 4,624,152, 7.9%)
Calendar year									
2011	16.1	19.2	21.0	1.6	2.2	13.0	17.3	3.5	8.7
2012	16.0	19.9	22.0	1.2	1.8	13.2	17.3	3.4	10.2
2013	15.3	19.8	22.4	1.2	2.3	12.2	16.9	3.4	9.7
2014	11.9	15.1	19.1	0.9	1.9	8.8	13.6	2.3	7.0
2015	13.7	16.8	21.1	0.9	2.5	9.6	15.7	2.6	7.9
2016	14.1	19.6	22.2	0.8	2.8	8.7	14.6	1.7	7.5
2017	12.6	18.1	20.7	1.0	2.8	8.5	14.8	1.3	7.5
2018	11.0	18.7	21.2	0.7	3.6	8.7	15.2	1.8	7.8
2019	11.1	16.3	20.9	0.3	4.8	8.5	14.3	2.3	7.9
Calendar quarter									
1	17.1	22.4	24.1	1.6	4.0	13.8	20.8	4.5	11.3
2	4.6	8.0	9.7	0.5	0.9	3.5	6.3	1.1	2.7
3	3.0	6.1	7.9	0.2	0.6	2.0	5.7	0.3	1.0
4	15.4	18.6	22.6	1.3	4.7	11.1	17.0	3.1	9.3
HHS region									
Region 1—Boston	11.0	18.9	23.0	0.7	2.7	7.8	8.8	1.3	7.4
Region 2—New York	7.6	13.0	18.9	1.8	3.0	8.1	14.9	4.5	6.9
Region 3—Philadelphia	12.1	16.1	21.4	0.9	3.5	9.8	15.5	3.6	7.8
Region 4—Atlanta	14.7	17.9	20.3	0.6	2.7	10.1	13.7	1.2	7.5
Region 5—Chicago	11.7	22.8	22.2	1.1	3.4	9.6	19.8	4.6	7.5
Region 6—Dallas	16.1	17.6	21.1	1.2	3.7	12.5	19.5	3.2	9.6
Region 7—Kansas City	15.4	21.0	22.1	1.3	3.9	11.2	19.5	1.1	8.0
Region 8—Denver	13.5	22.1	25.0	0.8	3.3	10.5	17.6	1.2	9.4
Region 9—San Francisco	10.9	22.1	21.1	1.0	2.0	9.0	16.0	1.0	7.4
Region 10—Seattle	9.4	25.4	23.6	0.5	2.5	8.5	14.0	2.1	7.8
Unknown	14.8	18.2	22.1	0.6	3.4				
Age group									
0	22.9	22.6	24.6	10.2	13.2				
1	19.0	17.8	21.1	4.0	8.8				
2	17.1	17.2	20.1	3.6	6.8				
3	12.6	15.2	16.9	1.4	4.5				
4	8.3	11.5	13.1	1.0	2.9				
5–9	3.0	5.6	7.8	0.5	1.1				
10–14	0.7	3.9	3.4	0.1	0.3				
15–17	0.4	3.9	3.2	0.1	0.2				
18–30	0.7	5.9	4.7	0.1	0.4				
31–40	0.7	3.2	4.7	0.1	0.4				
41–50	0.6	1.6	2.1	0.2	0.6				
51–64	0.8	1.1	1.6	0.1	0.8				
> = 65	1.3	1.4	1.0	0.2	1.6				
Clinical setting									
Inpatient visit	4.7	12.3	10.6	0.8	3.1				
Emergency visit	11.6	15.7	17.2	3.1	6.0				
Outpatient visit	14.2	19.9	22.1	0.8	2.2				
Indication for testing									
Severe complication	6.4	16.3	18.1	0.5	3.8				
LRTI	37.5	35.3	44.4	4.6	14.1				
Asthma exacerbation	3.5	2.6	4.8	1.2	2.6				
URTI	5.0	6.4	6.9	0.5	1.8				
Respiratory symptoms	6.7	13.3	13.2	0.8	1.8				
Others (e.g., flu/RSV infection)	9.1	17.4	20.6	0.4	1.5				

^*^In MarketScan RSV tests were identified using the “strict” definition, which required an RSV-specific test procedure code or an unspecific procedure code that was accompanied by a diagnosis indicative of respiratory illness (see Additional file 1: Table S1). Positivity was measured as the proportion of RSV tests accompanied by diagnoses codes on laboratory claims or adjacent medical encounters. Positivity for tests in NREVSS was directly extracted from recorded test results

MarketScan: MarketScan Commercial Claims and Medicare Supplemental Databases; NREVSS: National Respiratory and Enteric Virus Surveillance System; RSV: respiratory syncytial virus; PCR: polymerase chain reaction; HHS region: the U.S. Department of Health and Human Services region LRTIs: lower respiratory tract infections; URTIs: upper respiratory tract infections

**Fig. 4:**
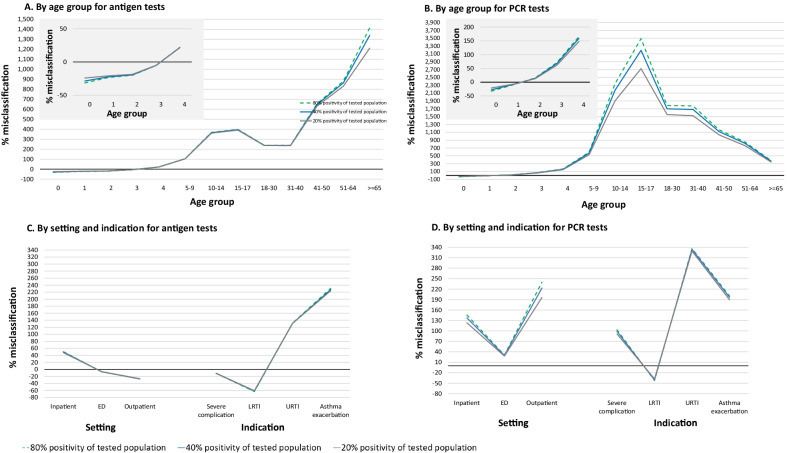
Impact of using pooled positivity from NREVSS on misclassification of RSV incidence for subpopulations

When using the average NREVSS positivity to correct RSV incidence for untested ARIs, we noted pronounced deviations from incidence estimates derived from stratum-specific positivity rates. For example, because we found highest antigen test positivity among infants, using the antigen test positivity from NREVSS that combines all age groups resulted in a − 32% underestimate of RSV incidence rates among infants below 1 year of age and a 1432% overestimate for patients 65 years of age and older if we assumed that untested population had the same positivity as the tested population (Fig. 4). Assuming the untested population had 10 times lower positivity than the tested population, the magnitude of misclassification decreased to − 18% for infants below 1 year of age and a 1017% for patients 65 years of age or older. Likewise, using NREVSS test positivity, which does not specify the setting, RSV inpatient incidences were overestimated by 50% or 147% based on antigen or PCR tests, respectively. Misclassification was reduced to 44.5% or 102% if we assumed that the positivity of the untested population was 10% of the tested population.

## Discussion

Large claims databases such as MarketScan are commonly used to derive population-based RSV incidence estimates. To correct for undertesting in clinical practice and undercoding in the claims process, resulting in ARI encounters without identified pathogen, researchers employ laboratory-based positivity rates such as those available within NREVSS. One critical assumption for the validity of this approach is that positivity is constant across all tested patients, or the population from which positivity results are obtained is representative of the population in which RSV incidence is measured. For example, if NREVSS positivity rates are used to estimate RSV-related LRTIs requiring hospital admission, the most common focus of positivity-based corrections [12–15], the pooled positivity from NREVSS would have to be applicable to this population.

Using MarketScan and NREVSS from 2011 to 2019, we examined the databases’ comparability in the epidemiology of RSV tests and test positivity across calendar years, quarters, regions, and test types. We further evaluated information only available in claims (i.e., age, clinical setting, and indication for testing) to gain insight in the possible composition of patients whose test data contribute to NREVSS. Our data, illustrating the variation in testing pattern and positivity, highlight the importance of generalizability assumptions when using NREVSS positivity rates to correct real-world encounter data to derive population-based incidence estimates. Two key findings of our study are noteworthy.

First, we noted pronounced variation in RSV testing pattern. As expected, both NREVSS and MarketScan showed seasonal differences in RSV testing and shifting preferences toward PCR tests over time. Of note, PCR tests accounted for 37.2% of all tests in MarketScan and nearly double of that in NREVSS (65.3%), which suggests differences in RSV test capture across these data sources. Possible reasons for such differences could be inherent in the types of laboratories that volunteer to contribute to NREVSS (e.g., hospital-affiliated labs may analyze more PCR tests), differential capture of test types in MarketScan (e.g., tests for hospitalized patients may be obscured in capitated reimbursement claims), or differences in the source population [28, 29]. Because of lower test sensitivity [18], we noted only a small proportion of adults with antigen tests in MarketScan, though a survey of NREVSS participating laboratories showed more than 37% of laboratories used antigen tests for adults [17].

Importantly, children less than 5 years of age contributed close to two thirds of all RSV tests in MarketScan, and more than three quarters of tests originated from outpatient care, which is consistent with our findings for testing indications including predominantly URTIs and respiratory symptoms. These findings are also consistent with previous prospective studies suggesting that RSV infections requiring medical care are most prominent among young children and are predominantly managed in the outpatient setting [11].

Keeping this description of the testing population in mind, our second finding related to test positivity is particularly relevant.

In MarketScan, we noted substantial variation in test positivity across age groups (age 0–4: 19.8%, age 5–17: 1.8% and adults: 0.7%), clinical settings (inpatient 4.7%, ED 11.6%, outpatient 14.2%), and indications for testing (range 5.0–35.9%). Our test positivity among children < 5 years (19.8%) was similar to a prospective study (18.1%) where all children < 5 years with ARIs were tested [11], suggesting relatively high positivity among children with ARIs but not tested for RSV. Both MarketScan and NREVSS also showed variation in positivity across regions. Furthermore, both databases had higher antigen positivity (NREVSS 16.0%, MarketScan 21.2%) compared to PCR positivity (NREVSS 7.9%, MarketScan 3.1%), an expected finding that has prompted the CDC to introduce different positivity thresholds to define RSV season onset (10% for antigen tests and 3% for PCR tests) [16, 19]. Of note, these differences in positivity across test types are largely attributable to differences in the population for who the tests are ordered and not the sensitivity of the test (e.g., PCR may be ordered after a negative RSV antigen test [17]). This further corroborates the finding that the overall RSV test positivity obtained from any data source is greatly dependent on the tested source population and thus, detail about the clinical characteristics of patients is critical if positivity results were used for inferences about the overall RSV disease burden.

Thus, NREVSS-based correction for RSV-under-ascertainment based on routinely-collected healthcare data may have some fallacies. For example, given the inclusion of an unknown proportion of tests from adults included in NREVSS, incidence estimates for infants will be underestimated. Likewise, use of the predominantly outpatient-originating test positivity rates to make inferences for inpatient RSV-LRTI, will likely result in inflated inpatient RSV incidence estimates. If NREVSS-based incidence corrections are applied for LRTI regardless of setting, the result is likely underestimated.

This is to our knowledge the first study using billing records to assess RSV testing practice and test positivity. We acknowledge some limitations of our study. First, while the MarketScan data supports detailed assessments across clinically-relevant strata, capitated payments may have obscured some ordered tests, especially in the inpatient setting. The proportion of medical encounters with RSV diagnoses for which we found an RSV laboratory test claim in MarketScan was 35% of inpatient RSV diagnoses and 55% for outpatient diagnosis, suggesting that some tests are not submitted for reimbursement. Thus, the reported frequencies for RSV tests are likely underestimates and the proportional distribution of tests across settings and patients may be affected by differential claims capture. This however, will not affect the measured positivity rates, because this calculation was based on unobscured RSV test claims. Second, our calculation of corrected RSV incidence rates based on NREVSS positivity rates used a simple computational approach, which relies on the assumption that test positivity is the same between tested ARIs and untested ARIs. Previous studies have used more sophisticated regression modeling instead, though both approaches rely on the assumption that test positivity is applicable to the patient population that has uncoded ARIs [12, 14, 15]. Thus, the reported direction of bias in our analysis still holds.

Third, RSV coding is known to have a high specificity but lower sensitivity [13] and therefore, we might have underestimated test positivity in MarketScan, though the average positivity in MarketScan was higher than in NREVSS. Fourth, because MarketScan only includes claims data from supplemental private insurance of Medicare beneficiaries, our results presented for patients > 65 years of age need to be interpreted with caution. These data do not represent the general Medicare population in the US and because supplemental insurance only covers costs that were not entirely reimbursed by Medicare, both testing claims or medical encounters for RSV may have been missed. Lastly, our presented comparisons reflect testing practices and positivity obtained from a privately insured population in MarketScan. Testing practices and positivity rates may vary in a more diverse population including also publicly and uninsured patients, which may be included in NREVSS. However, the presented differences across age groups and clinical settings regarding clinical decision-making on whether to order a test and test positivity are expected to be present across all health insurance types, highlighting the importance when making inferences from aggregate NREVSS data for specific patient populations. Further research should replicate our study among Medicaid and Medicare beneficiaries for more comprehensive comparisons to further elucidate the relationship between patient characteristics and testing practices and RSV test results.

## Conclusion

RSV test positivity estimates are dependent on the characteristics of patients for who those tests were ordered, and RSV testing practice and test positivity vary considerably across patient groups. Claims for RSV tests originated predominantly from outpatient encounters of infants and young children. Both groups also had the highest test positivity suggesting limited generalizability to inpatient encounters and adults. NREVSS-based correction for RSV-under-ascertainment in routinely-collected healthcare data may underestimate the true RSV incidence among young children and may overestimate rates among adults and inpatient admissions. Demographic and clinical detail on testing practice and positivity should be considered for RSV test positivity corrections to improve the accuracy of population-based RSV incidence estimates when adjusting for lack of testing.

## Supplementary Information

**Additional file 1. ** Supplementary Methods.** Table S1.** Current Procedure Terminology codes for respiratory syncytial virus tests. **Table S2.** Diagnosis codes to measure respiratory syncytial virus infection (RSV) and infection severity. **Table S3.** Crude distribution of RSV tests in MarketScan and NREVSS (2011–2019) databases. **Table S4.** Crude distribution of RSV tests in MarketScan Commercial Claims and Medicare Supplemental Databases and the National Respiratory and Enteric Virus Surveillance System (2011–2019), stratified by test type.

## Data Availability

Patient data that support the findings of this study are available from Truven Health Analytics Inc. (https://www.ibm.com/watson-health/about/truven-health-analytics). However, these data were used under license for the current study, and so are not publicly available. Data are however available from the author (Almut Winterstein, email: almut@ufl.edu, address 1225 Center Drive, PO Box 100496, Gainesville, Florida, 32611-0496, telephone: (352) 273-6268, fax: (352)-273-6270) upon reasonable request and with permission of IBM MarketScan.

## Abbreviations

RSV: Respiratory syncytial virus
CDC: The Centers for Disease Control and Prevention
NREVSS: CDC’s National Respiratory and Enteric Virus Surveillance System
LRTI: Lower respiratory tract infections
PCR: Polymerase chain reaction
ARI: Acute respiratory infections
