# Supplementary material for: RSV testing practice and positivity by patient demographics in the United States: integrated analyses of MarketScan and NREVSS databases
Source: BMC Infect Dis. 2022 Aug 8;22:681. doi: 10.1186/s12879-022-07659-x (PMC9360654; doi:10.1186/s12879-022-07659-x)
Supplement: Supplementary file 1 — Additional file 1. Supplementary Methods. Table S1. Current Procedure Terminology codes for respiratory syncytial virus tests. Table S2. Diagnosis codes to measure respiratory syncytial virus infection (RSV) and infection severity. Table S3. Crude distribution of RSV tests in MarketScan and NREVSS (2011–2019) databases. Table S4. Crude distribution of RSV tests in MarketScan Commercial Claims and Medicare Supplemental Databases and the National Respiratory and Enteric Virus Surveillance System (2011–2019), stratified by test type. [file 12879_2022_7659_MOESM1_ESM.docx]

**Supplementary Materials**

**Supplementary Methods**

**Supplementary Tables**

**Table S1.** Current Procedure Terminology codes for respiratory syncytial virus tests

**Table S2.** Diagnosis codes to measure respiratory syncytial virus infection (RSV) and infection severity

**Table S3.** Crude distribution of RSV tests in MarketScan and NREVSS (2011-2019) databases.

**Table S4.** Crude distribution of RSV tests in MarketScan Commercial Claims and Medicare Supplemental Databases and the National Respiratory and Enteric Virus Surveillance System (2011-2019), stratified by test type

**Supplementary Methods**

Assume we have 100 patients with ARIs, 20 of them have RSV test claims and 2 have positive tests, thus the positivity rate is 2/20=10%. We find 6 ARIs with RSV diagnoses in total, suggesting that at least 4 RSV tests were obscured in the claims data. The remaining ARIs with no information on the pathogen represent a combination of ARIs for which no tests were ordered and ARIs for which tests were ordered but also obscured in the claims data, thus appearing to be negative.

To obtain the number of truly untested ARIs, we estimate the number of ARIs without RSV tests based on the detected positivity. This assumes that the RSV positivity rate among those ARIs not tested is the same as among those tested. With this assumption, the number of tests needed to have 6 RSV encounters should be 6/10%=60 tests. Thus, the number of ARIs without RSV tests is 100-60=40.

We then used this number of ARIs without RSV tests (40 instead of 80) to correct the overall RSV incidence, applying a range of RSV positivity to the untested ARIs. For the untested population, we varied positivity rates to be 10%, 20%, 40%, 60%, 80% or 100% of positivity in the tested population, i.e., with a positivity rate of 10% in the tested population, applied positivity for the untested population ranged from 1% (10%*10%) to 10% (100%*10%). For example, using the 8% (80%*10%) positivity for the untested population, the corrected incidence is 6+40*8%=9.2 RSV cases over 100 ARIs.

From equations 1 and 2 in the method, we have

$$\%misclassification=\frac{\bar{ARI}_{RSV} using MarketScan-\bar{ARI}_{RSV} using NREVSS}{{ARI}_{RSV}+{ARI}_{RSV*} using MarketScan}*100$$

Let’s take age 0, using PCR test for correction as an example.

| **Assumed positivity of untested patients** | **Data obtained from MarketScan and NREVSS** | | | | | **Corrected RSV encounters** | | **% misclassification** |
| --- | --- | --- | --- | --- | --- | --- | --- | --- |
|  | ARI cases  (A) | Coded RSV (B) | MarketScan positivity (C) | Average NREVSS positivity (D) | Untested RSV (E ) | Using MarketScan positivity (F) | Using average NREVSS positivity (G) |  |
| 100% of C or D | 12,697,095 | 255,971 | 13.2 | 7.9 | = A-B/C =  10,757,921 | = B+ E*C/100  = 1,676,017 | B+E*D/100 = 1,105,847 | =(G-F)*100/F =  -34.0% |
| 10% of C or D |  |  |  |  |  | = B+ E*C*0.1/100= 397,976 | B+E*D*0.1/100= 340,959 | =(G-F)*100/F =  -14.3% |

**Supplementary Tables**

**Table S1.** Current Procedure Terminology codes for respiratory syncytial virus tests

| **CPT Codes** | **Description** | **Test Type** |
| --- | --- | --- |
| 87807 | Infectious agent antigen detection by immunoassay with direct optical observation; **respiratory syncytial virus** | Rapid Antigen |
| 87420 | Infectious agent antigen detection by immunoassay technique, (eg, enzyme immunoassay [EIA], enzyme-linked immunosorbent assay [ELISA], immunochemiluminometric assay [IMCA]) qualitative or semiquantitative, multiple-step method; **respiratory syncytial virus** |  |
| 86756 | Antibody; **respiratory syncytial virus**  **(Not recommended for clinical practice but rather for research purposes, excluded in the very strict definition but retained in the strict definition)** | Antibody |
| 87280 | Infectious agent antigen detection by immunofluorescent technique; **respiratory syncytial virus** | Immunofluorescent |
| 87300 | Infectious agent antigen detection by immunofluorescent technique, polyvalent for multiple organisms, each polyvalent antiserum  **(test claims required to have a diagnosis code related to respiratory disease or symptoms for the strict definition but not for the broad definition)** |  |
| 87798 | Infectious agent detection by nucleic acid (DNA or RNA), not otherwise specified; amplified probe technique, each organism  **(test claims required to have a diagnosis code related to respiratory disease or symptoms for the strict definition but not for the broad definition)** | PCR |
| 87634 | Infectious agent detection by nucleic acid (DNA or RNA); **respiratory syncytial virus**, amplified probe technique |  |
| 87631 | Infectious agent detection by nucleic acid (DNA or RNA); respiratory virus (eg, adenovirus, influenza virus, coronavirus, metapneumovirus, parainfluenza virus, **respiratory syncytial virus**, rhinovirus), includes multiplex reverse transcription, when performed, and multiplex amplified probe technique, multiple types or subtypes, 3-5 targets |  |
| 87632 | Infectious agent detection by nucleic acid (DNA or RNA); respiratory virus (eg, adenovirus, influenza virus, coronavirus, metapneumovirus, parainfluenza virus, **respiratory syncytial virus**, rhinovirus), includes multiplex reverse transcription, when performed, and multiplex amplified probe technique, multiple types or subtypes, 6-11 targets |  |
| 87633 | Infectious agent detection by nucleic acid (DNA or RNA); respiratory virus (eg, adenovirus, influenza virus, coronavirus, metapneumovirus, parainfluenza virus, **respiratory syncytial virus,** rhinovirus), includes multiplex reverse transcription, when performed, and multiplex amplified probe technique, multiple types or subtypes, 12-25 targets |  |
| 0100U | Respiratory pathogen, multiplex reverse transcription and multiplex amplified probe technique, multiple types or subtypes, 21 targets (adenovirus, coronavirus 229E, coronavirus HKU1, coronavirus NL63, coronavirus OC43, human metapneumovirus, human rhinovirus/enterovirus, influenza A, including subtypes H1, H1-2009, and H3, influenza B, parainfluenza virus 1, parainfluenza virus 2, parainfluenza virus 3, parainfluenza virus 4, **respiratory syncytial virus**, Bordetella parapertussis [IS1001], Bordetella pertussis [ptxP], Chlamydia pneumoniae, Mycoplasma pneumoniae) |  |
| 0099U | Respiratory pathogen, multiplex reverse transcription and multiplex amplified probe technique, multiple types or subtypes, 20 targets (adenovirus, coronavirus 229E, coronavirus HKU1, coronavirus, coronavirus OC43, human metapneumovirus, influenza A, influenza A subtype, influenza A subtype H3, influenza A subtype H1-2009, influenza, parainfluenza virus, parainfluenza virus 2, parainfluenza virus 3, parainfluenza virus 4, human rhinovirus/enterovirus, **respiratory syncytial virus**, Bordetella pertussis, Chlamydophila pneumonia, Mycoplasma pneumoniae) |  |
| 0098U | Respiratory pathogen, multiplex reverse transcription and multiplex amplified probe technique, multiple types or subtypes, 14 targets (adenovirus, coronavirus, human metapneumovirus, influenza A, influenza A subtype H1, influenza A subtype H3, influenza A subtype H1-2009, influenza B, parainfluenza virus, human rhinovirus/enterovirus, **respiratory syncytial virus**, Bordetella pertussis, Chlamydophila pneumoniae, Mycoplasma pneumoniae) |  |
| 87637 | Infectious agent detection by nucleic acid (DNA or RNA); severe acute respiratory syndrome coronavirus 2 (SARS-CoV-2) (Coronavirus disease [COVID-19]), influenza virus types A and B, and **respiratory syncytial virus**, multiplex amplified probe technique | PCR  Do not appear in our dataset |
| 0241U | Infectious disease (viral respiratory tract infection), pathogen-specific RNA, 4 targets (severe acute respiratory syndrome coronavirus 2 [SARS-CoV-2], influenza A, influenza B, **respiratory syncytial virus [RSV]**), upper respiratory specimen, each pathogen reported as detected or not detected |  |
| 0115U | **Respiratory infectious agent** detection by nucleic acid (DNA and RNA), **18 viral types and subtypes** and 2 bacterial targets, amplified probe technique, including multiplex reverse transcription for RNA targets, each analyte reported as detected or not detected |  |
| 0151U | Infectious disease (bacterial or **viral respiratory tract infection**), pathogen specific nucleic acid (DNA or RNA), 33 targets, real-time semi-quantitative PCR, bronchoalveolar lavage, sputum, or endotracheal aspirate, detection of 33 organismal and antibiotic resistance genes with limited semi-quantitative results |  |
| 0202U | Infectious disease (bacterial or **viral respiratory tract infection**), pathogen-specific nucleic acid (DNA or RNA), 22 targets including severe acute respiratory syndrome coronavirus 2 (SARS-CoV-2), qualitative RT-PCR, nasopharyngeal swab, each pathogen reported as detected or not detected |  |
| 0223U | Infectious disease (bacterial or **viral respiratory tract infection**), pathogen-specific nucleic acid (DNA or RNA), 22 targets including severe acute respiratory syndrome coronavirus 2 (SARS-CoV-2), qualitative RT-PCR, nasopharyngeal swab, each pathogen reported as detected or not detected |  |
| 0225U | Infectious disease (bacterial or **viral respiratory tract infection**) pathogen-specific DNA and RNA, 21 targets, including severe acute respiratory syndrome coronavirus 2 (SARSCoV-2), amplified probe technique, including multiplex reverse transcription for RNA targets, each analyte reported as detected or not detected |  |
| 87140 | Culture, typing; immunofluorescent method, each antiserum  **(test claims required to have a diagnosis code related to respiratory disease or symptoms for the strict definition but not for the broad definition)** | Virus isolation/culture |
| 87253 | Virus isolation; tissue culture, additional studies or definitive identification (eg, hemabsorption, neutralization, immunofluorescence stain), each isolate  **(test claims required to have a diagnosis code related to respiratory disease or symptoms for the strict definition but not for the broad definition)** |  |
| 87252 | Virus isolation; tissue culture inoculation, observation, and presumptive identification by cytopathic effect  **(test claims required to have a diagnosis code related to respiratory disease or symptoms for the strict definition but not for the broad definition)** |  |
| 87254 | Virus isolation; centrifuge enhanced (shell vial) technique, includes identification with immunofluorescence stain, each virus  **(test claims required to have a diagnosis code related to respiratory disease or symptoms for the strict definition but not for the broad definition)** |  |
| 87255 | Virus isolation; including identification by non-immunologic method, other than by cytopathic effect (eg, virus specific enzymatic activity)  **(test claims required to have a diagnosis code related to respiratory disease or symptoms for the strict definition but not for the broad definition)** |  |

**Table S2.** Diagnosis codes to measure respiratory syncytial virus infection (RSV) and infection severity.

|  | **ICD-9-CM** |  |
| --- | --- | --- |
| **RSV infection** | 0796, 4801, 46611 | J121, J210, J205, B974 |
| **Septicemia /sepsis** | 0545, 449, 77181, 7907, 99591, 99592, 0031, 0202, 0223, 0362, 0380, 0381, 03810, 03811, 03812, 03819, 0382, 0383, 03840, 03841, 03842, 03843, 03844, 03849, 0388, 0389 | A021, A021, A207, A227, A267, A327, A392, A393, A394, A400, A400, A401, A403, A408, A409, A409, A4101, A4102, A411, A412, A413, A414, A4151, A4152, A4153, A4159, A4181, A4189, A419, A427, A5486, B007, B377, I76, P360, P3610, P3619, P362, P3630, P3639, P364, P365, P368, P369, R6520, R6521, R7881 |
| **Respiratory failure/distress** | 51881, 51882, 51883, 51884, 769, 7706, 77089 | J80, R06.03, J96, P22 |
| **Low respiratory tract infection (LRTI):** Pneumonia | 00322, 0203, 0204, 0205, 0212,  0221, 0310, 0391, 0521, 0551, 0730, 0730, 0830, 1124, 1140, 1144, 1145, 11505, 11515, 11515, 11595, 11595, 1304, 1363, 480-486, 4870, 5171 | A0222, A202, A212, A221, A221, A310, A3701, A3711, A3781, A3791, A420, A430, A481, A70, A78, B012, B052, B250, B371, B380, B381, B382, B390, B391, B392, B395, B399, B440, B583, B59, B7781, J1000, J1001, J1108, J12-J18 |
| **LRTI:** Bronchiolitis/bronchitis | 466, 490 | J40, J20, J210, J21 |
| **Asthma** with acute exacerbation | 49302, 49312, 49322, 49392 | J4521, J4531, J4541, J4551, J441, J45901 |
| **Upper respiratory tract infection** (e.g., pharyngitis, sinusitis, tonsillitis, laryngitis, tracheitis, epiglottitis, other disease of nasal cavity and sinuses, **otitis)** | 0320, 0321, 0322, 0323, 0340, 460-465, 473, 474, 4781, 78491,  3801-3802, 3810-3814, 382 | A360, A361, A362, A3689, J00-J06, J32, J340, J341, J3481, J3489, J349, J35, H60, H6190, H6191, H6192, H6193, H624, H628X1, H628X2, H628X3, H628X9, H65-H67 |
| **Respiratory Symptoms** (cough, abnormalities of breathing, pain in throat and chest, other symptoms and signs involving the circulatory and respiratory system, fever unspecified) | 79902, 79901, 7991, 786, 78060 | R05, R06, R07, R09, R509 |

**Table S3.** Crude distribution of RSV tests in MarketScan and NREVSS (2011-2019) databases.

|  | **RSV testing definitions - MarketScan (N, %)** | | | **NREVSS (N, %)**  **(N=7 079 301)** |
| --- | --- | --- | --- | --- |
| **Variables** | **Very strict (N=1 206 704)** | **Strict (N=1 471 777)** | **Broad (N=3 684 704)** |  |
| **Calendar year** |  |  |  |  |
| 2011 | 112 804 (9.3) | 147 365 (10.0) | 372 464 (10.1) | 674 257 (9.5) |
| 2012 | 133 089 (11.0) | 184 004 (12.5) | 506 082 (13.7) | 639 542 (9.0) |
| 2013 | 122 502 (10.2) | 155 885 (10.6) | 487 688 (13.2) | 621 183 (8.8) |
| 2014 | 136 804 (11.3) | 172 282 (11.7) | 452 432 (12.3) | 532 196 (7.5) |
| 2015 | 101 781 (8.4) | 122 261 (8.3) | 317 239 (8.6) | 659 239 (9.3) |
| 2016 | 122 810 (10.2) | 145 053 (9.9) | 364 000 (9.9) | 838 913 (11.9) |
| 2017 | 135 103 (11.2) | 158 056 (10.7) | 375 723 (10.2) | 937 321 (13.2) |
| 2018 | 160 961 (13.3) | 183 863 (12.5) | 394 825 (10.7) | 1 041 624 (14.7) |
| 2019 | 180 850 (15.0) | 203 008 (13.8) | 414 251 (11.2) | 1 135 026 (16.0) |
| **Calendar quarter** |  |  |  |  |
| Quarter 1 | 561608 (46.5) | 646141 (43.9) | 1177932 (32.0) | 2995440 (42.3) |
| Quarter 2 | 148541 (12.3) | 202572 (13.8) | 746810 (20.3) | 1293009 (18.3) |
| Quarter 3 | 90393 (7.5) | 139740 (9.5) | 715197 (19.4) | 789840 (11.2) |
| Quarter 4 | 406162 (33.7) | 483324 (32.8) | 1044765 (28.3) | 2001012 (28.3) |
| **HHS region** |  |  |  |  |
| Region 1 – Boston | 22 872 (1.9) | 34 767 (2.4) | 105 462 (2.9) | 344 602 (4.9) |
| Region 2 – New York | 87 011 (7.2) | 116 356 (7.9) | 463 892 (12.6) | 644 095 (9.1) |
| Region 3 – Philadelphia | 76 575 (6.3) | 95 336 (6.5) | 313 624 (8.5) | 540 713 (7.6) |
| Region 4 – Atlanta | 330 960 (27.4) | 370 442 (25.2) | 896 490 (24.3) | 1 015 649 (14.3) |
| Region 5 – Chicago | 159 479 (13.2) | 213 729 (14.5) | 482 104 (13.1) | 1 433 596 (20.3) |
| Region 6 – Dallas | 293 926 (24.4) | 332 430 (22.6) | 690 343 (18.7) | 987 792 (14.0) |
| Region 7 – Kansas City | 61007 (5.1) | 71565 (4.9) | 126 648 (3.4) | 338 078 (4.8) |
| Region 8 – Denver | 28 701 (2.4) | 41 253 (2.8) | 83 340 (2.3) | 484 185 (6.8) |
| Region 9 – San Francisco | 55 913 (4.6) | 81795 (5.6) | 268 449 (7.3) | 806 913 (11.4) |
| Region 10 – Seattle | 20 715 (1.7) | 35 059 (2.4) | 75 360 (2.0) | 483 678 (6.8) |
| Unknown | 69 545 (5.8) | 79 045 (5.4) | 178 992 (4.9) |  |
| **Type of RSV tests** |  |  |  |  |
| PCR | 341 085 (28.3) | 548 091 (37.2) | 2 331 073 (63.3) | 4 624 153 (65.3) |
| Viral isolation/culture | 0 (0) | 68 930 (4.7) | 498 504 (13.5) | 471 707 (6.7) |
| Antigen | 830 596 (68.8) | 820 426 (55.7) | 820 884 (22.3) | 1 983 441 (28.0) |
| Antibody | 35 023 (2.9) | 34 330 (2.3) | 34 243 (0.9) | 0 |
| **Age group** |  |  |  |  |
| 0 | 456 976 (37.9) | 477 387 (32.4) | 487 683 (13.2) |  |
| 1 | 260 432 (21.6) | 272 390 (18.5) | 279 392 (7.6) |  |
| 2 | 113 034 (9.4) | 122 267 (8.3) | 127 394 (3.5) |  |
| 3 | 55 235 (4.6) | 63 818 (4.3) | 68 172 (1.9) |  |
| 4 | 30 982 (2.6) | 38 617 (2.6) | 42 637 (1.2) |  |
| 5-9 | 59 622 (4.9) | 90 319 (6.1) | 108 905 (3.0) |  |
| 10-14 | 27 092 (2.2) | 53 413 (3.6) | 78 423 (2.1) |  |
| 15-17 | 13 552 (1.1) | 29 164 (2.0) | 93 310 (2.5) |  |
| 18-30 | 41 083 (3.4) | 76 462 (5.2) | 939 193 (25.5) |  |
| 31-40 | 35 340 (2.9) | 61 306 (4.2) | 57 4745 (15.6) |  |
| 41-50 | 37 047 (3.1) | 64 366 (4.4) | 419 292 (11.4) |  |
| 51-64 | 63 001 (5.2) | 101 416 (6.9) | 415 951 (11.3) |  |
| >=65 | 13 308 (1.1) | 20 852 (1.4) | 49 607 (1.3) |  |
| **Clinical setting** |  |  |  |  |
| Inpatient visit | 32 990 (2.7) | 46 023 (3.1) | 60 464 (1.6) |  |
| Emergency visit^a^ | 241 790 (20.0) | 273 127 (18.6) | 306 439 (8.3) |  |
| Outpatient visit^a^ | 931 924 (77.2) | 1 152 627 (78.3) | 3 317 801 (90.0) |  |
| **Indications for testing** |  |  |  |  |
| Severe complication | 14 390 (1.2) | 21 192 (1.4) | 21 148 (0.6) |  |
| LRTI | 304 748 (25.3) | 331 609 (22.5) | 331 373 (9.0) |  |
| Asthma exacerbation | 13 719 (1.1) | 15 981 (1.1) | 15 977 (0.4) |  |
| URTI | 399 391 (33.1) | 469 696 (31.9) | 469 121 (12.7) |  |
| Respiratory symptoms | 276 290 (22.9) | 410 035 (27.9) | 409 520 (11.1) |  |
| Others (e.g., flu/RSV infection) | 198 166 (16.4) | 223 264 (15.2) | 2 437 565 (66.2) |  |
| **RSV diagnosis on test claims** |  |  |  |  |
| Yes | 164 357 (13.6) | 165 831 (11.3) | 1. 059 (4.5) |  |

^a^ Nearly 2% of tests in outpatient setting was overlapped with an admission within 3 days.

Abbreviations – MarketScan: MarketScan Commercial Claims and Medicare Supplemental Databases; NREVSS: National Respiratory and Enteric Virus Surveillance System; RSV: respiratory syncytial virus; PCR: polymerase chain reaction; HHS region: the U.S. Department of Health and Human Services region LRTIs: lower respiratory tract infections; URTIs: upper respiratory tract infections.

**Table S4.** Crude distribution of RSV tests in MarketScan Commercial Claims and Medicare Supplemental Databases and the National Respiratory and Enteric Virus Surveillance System (2011-2019), stratified by test type

|  | **MarketScan* (N, %)** | | | |  | **NREVSS (N, %)** | | |
| --- | --- | --- | --- | --- | --- | --- | --- | --- |
| **Variables** | **Antibody (N=34 330)** | **Antigen (N=820 426)** | **Viral culture (N=68 930)** | **PCR (N=548 091)** |  | **Antigen (N=1 983 441)** | **Viral culture (N=471 707)** | **PCR (N=4 624 152)** |
| **Calendar year** |  |  |  |  |  |  |  |  |
| 2011 | 6 614 (19.3) | 103 912 (12.7) | 12 926 (18.8) | 23 913 (4.4) |  | 402 383 (20.3) | 107981 (22.9) | 163 893 (3.5) |
| 2012 | 7 165 (20.9) | 123 467 (15.0) | 12 106 (17.6) | 41 266 (7.5) |  | 359 045 (18.1) | 91769 (19.5) | 188 728 (4.1) |
| 2013 | 4 915 (14.3) | 97 454 (11.9) | 10 983 (15.9) | 42 533 (7.8) |  | 275 506 (13.9) | 70098 (14.9) | 275 579 (6.0) |
| 2014 | 4 280 (12.5) | 97 914 (11.9) | 10 041 (14.6) | 60 047 (11.0) |  | 176 952 (8.9) | 51082 (10.8) | 304 162 (6.6) |
| 2015 | 2 586 (7.5) | 72 782 (8.9) | 5 153 (7.5) | 41 740 (7.6) |  | 171 897 (8.7) | 40071 (8.5) | 447 271 (9.7) |
| 2016 | 2 644 (7.7) | 82 923 (10.1) | 4 761 (6.9) | 54 725 (10.0) |  | 167 762 (8.5) | 32066 (6.8) | 639 085 (13.8) |
| 2017 | 2 202 (6.4) | 85 178 (10.4) | 4 413 (6.4) | 66 263 (12.1) |  | 150 800 (7.6) | 26830 (5.7) | 759 691 (16.4) |
| 2018 | 2 145 (6.2) | 76 837 (9.4) | 4 860 (7.1) | 100 021 (18.2) |  | 141 615 (7.1) | 26887 (5.7) | 873 122 (18.9) |
| 2019 | 1 779 (5.2) | 79 959 (9.7) | 3 687 (5.3) | 117 583 (21.5) |  | 137 481 (6.9) | 24923 (5.3) | 972 622 (21) |
| **Calendar quarter** |  |  |  |  |  |  |  |  |
| 1 | 17 350 (50.5) | 408 115 (49.7) | 28 106 (40.8) | 192 570 (35.1) |  | 929 033 (46.8) | 191352 (40.6) | 1875 055 (40.5) |
| 2 | 3 883 (11.3) | 84 310 (10.3) | 12 836 (18.6) | 101 543 (18.5) |  | 336 002 (16.9) | 97060 (20.6) | 859 947 (18.6) |
| 3 | 2 069 (6.0) | 44 748 (5.5) | 9 960 (14.4) | 82 963 (15.1) |  | 177 290 (8.9) | 65 975 (14.0) | 546 575 (11.8) |
| 4 | 11 028 (32.1) | 283 253 (34.5) | 18 028 (26.2) | 171 015 (31.2) |  | 541 116 (27.3) | 117 320 (24.9) | 1342 576 (29.0) |
| **HHS region** |  |  |  |  |  |  |  |  |
| Region 1 - Boston | 419 (1.2) | 14 355 (1.7) | 3 127 (4.5) | 16 866 (3.1) |  | 147 590 (7.4) | 8 897 (1.9) | 188 115 (4.1) |
| Region 2 - New York | 1 928 (5.6) | 33 805 (4.1) | 12 286 (17.8) | 68 337 (12.5) |  | 112 873 (5.7) | 62 928 (13.3) | 468 294 (10.1) |
| Region 3 - Philadelphia | 1 981 (5.8) | 45 600 (5.6) | 5 372 (7.8) | 42 383 (7.7) |  | 167 182 (8.4) | 49 017 (10.4) | 324 514 (7) |
| Region 4 - Atlanta | 11 657 (34.0) | 242 825 (29.6) | 11 046 (16.0) | 104 914 (19.1) |  | 499 785 (25.2) | 75 170 (15.9) | 440 694 (9.5) |
| Region 5 - Chicago | 3 390 (9.9) | 92 899 (11.3) | 10 851 (15.7) | 106 589 (19.4) |  | 266 606 (13.4) | 103 969 (22) | 1063 021 (23) |
| Region 6 - Dallas | 8 427 (24.5) | 232 684 (28.4) | 13 337 (19.3) | 77 982 (14.2) |  | 306 394 (15.4) | 28 644 (6.1) | 652 754 (14.1) |
| Region 7 - Kansas City | 1 775 (5.2) | 43 923 (5.4) | 1 768 (2.6) | 24 099 (4.4) |  | 114 547 (5.8) | 34 901 (7.4) | 188 630 (4.1) |
| Region 8 - Denver | 420 (1.2) | 19 191 (2.3) | 936 (1.4) | 20 706 (3.8) |  | 85 547 (4.3) | 19 953 (4.2) | 378 685 (8.2) |
| Region 9 - San Francisco | 2 100 (6.1) | 36 531 (4.5) | 5 672 (8.2) | 37 492 (6.8) |  | 178 850 (9) | 35 450 (7.5) | 592 613 (12.8) |
| Region 10 - Seattle | 422 (1.2) | 11 273 (1.4) | 2 243 (3.3) | 21 121 (3.9) |  | 104 067 (5.2) | 52 778 (11.2) | 326 833 (7.1) |
| Unknown | 1 811 (5.3) | 47 340 (5.8) | 2 292 (3.3) | 27 602 (5.0) |  |  |  |  |
| **Age group** |  |  |  |  |  |  |  |  |
| 0 | 15 944 (46.4) | 394 374 (48.1) | 4 641 (6.7) | 62 428 (11.4) |  |  |  |  |
| 1 | 9 175 (26.7) | 220 306 (26.9) | 3 121 (4.5) | 39 788 (7.3) |  |  |  |  |
| 2 | 3 871 (11.3) | 91 669 (11.2) | 1 761 (2.6) | 24 966 (4.6) |  |  |  |  |
| 3 | 1 762 (5.1) | 40 774 (5.0) | 1 343 (1.9) | 19 939 (3.6) |  |  |  |  |
| 4 | 883 (2.6) | 19 893 (2.4) | 1 161 (1.7) | 16 680 (3.0) |  |  |  |  |
| 5-9 | 1 120 (3.3) | 25 148 (3.1) | 4 531 (6.6) | 59 520 (10.9) |  |  |  |  |
| 10-14 | 236 (0.7) | 6 104 (0.7) | 3 362 (4.9) | 43 711 (8.0) |  |  |  |  |
| 15-17 | 80 (0.2) | 2 185 (0.3) | 2 503 (3.6) | 24 396 (4.5) |  |  |  |  |
| 18-30 | 317 (0.9) | 5 042 (0.6) | 11 825 (17.2) | 59 278 (10.8) |  |  |  |  |
| 31-40 | 260 (0.8) | 4 631 (0.6) | 8 599 (12.5) | 47 816 (8.7) |  |  |  |  |
| 41-50 | 249 (0.7) | 3 685 (0.4) | 9 043 (13.1) | 51 389 (9.4) |  |  |  |  |
| 51-64 | 359 (1.0) | 5 335 (0.7) | 13 675 (19.8) | 82 047 (15.0) |  |  |  |  |
| >=65 | 74 (0.2) | 1 280 (0.2) | 3 365 (4.9) | 16 133 (2.9) |  |  |  |  |
| **Clinical setting** |  |  |  |  |  |  |  |  |
| Inpatient visit | 783 (2.3) | 11 753 (1.4) | 4 719 (6.8) | 28 768 (5.2) |  |  |  |  |
| Emergency visit | 9 995 (29.1) | 130 533 (15.9) | 9 202 (13.3) | 123 397 (22.5) |  |  |  |  |
| Outpatient visit | 23 552 (68.6) | 678 140 (82.7) | 55 009 (79.8) | 395 926 (72.2) |  |  |  |  |
| **Indication for testing** |  |  |  |  |  |  |  |  |
| Severe complication | 186 (0.5) | 4 524 (0.6) | 2 459 (3.6) | 14 023 (2.6) |  |  |  |  |
| LRTI | 10 846 (31.6) | 251 604 (30.7) | 9 274 (13.5) | 59 885 (10.9) |  |  |  |  |
| Asthma exacerbation | 313 (0.9) | 7 049 (0.9) | 501 (0.7) | 8 118 (1.5) |  |  |  |  |
| URTI | 11 016 (32.1) | 297 700 (36.3) | 25 912 (37.5) | 135 168 (24.7) |  |  |  |  |
| Respiratory symptoms | 7 396 (21.5) | 172 354 (21.0) | 18 408 (26.7) | 211 877 (38.7) |  |  |  |  |
| Others (e.g., flu/RSV infection) | 4 773 (13.4) | 97 195 (10.6) | 12 476 (18.1) | 119 020 (21.1) |  |  |  |  |
| **RSV diagnosis on test claims** |  |  |  |  |  |  |  |  |
| No | 29 156 (84.9) | 672 936 (82.0) | 68 082 (98.8) | 535 772 (97.8) |  |  |  |  |
| Yes | 5 174 (15.1) | 147 490 (18.0) | 848 (1.2) | 12 319 (2.2) |  |  |  |  |

(*) In MarketScan RSV tests were identified using the “strict” definition, which required an RSV-specific test procedure code or an unspecific procedure code that was accompanied by a diagnosis indicative of respiratory illness (see supplemental table S1). Positivity was measured as the proportion of RSV tests accompanied by diagnoses codes on laboratory claims or adjacent medical encounters. Positivity for tests in NREVSS was directly extracted from recorded test results.

Abbreviations – MarketScan: MarketScan Commercial Claims and Medicare Supplemental Databases; NREVSS: National Respiratory and Enteric Virus Surveillance System; RSV: respiratory syncytial virus; PCR: polymerase chain reaction; HHS region: the U.S. Department of Health and Human Services region LRTIs: lower respiratory tract infections; URTIs: upper respiratory tract infections.
